# A high-fat plus high-sucrose diet induces age-related macular degeneration in an experimental rabbit model

**DOI:** 10.1242/dmm.052015

**Published:** 2024-11-27

**Authors:** Yujiao Wang, Zhongping Lv, Yongjiang Chen, Xiaobo Cen, Hui Zhang, Danian Chen

**Affiliations:** ^1^ Department of Ophthalmology, Research Laboratory of Ophthalmology and Vision Sciences, Eye Research Institute, West China Hospital, Sichuan University, Chengdu 610041, China; ^2^ National Chengdu Center for Safety Evaluation of Drugs, West China-Frontier Pharma Tech Co., Ltd., Chengdu 610041, China; ^3^ Waterloo eye institute, School of Optometry and Vision Science, University of Waterloo, 200 University Ave. W., Waterloo, ON N2L 3G1, Canada

**Keywords:** Age-related macular degeneration, Normal-weight dyslipidemia, Drusen, Lipid droplets, dry AMD, wet AMD

## Abstract

Age-related macular degeneration (AMD) is a leading cause of blindness. Metabolic disorders and diets are risk factors. We compared lipid profiles and retinal phenotypes with long-term feeding of four diets in male Chinchilla rabbits. Animals were fed a normal diet (ND), high-fat diet (HFD), high-sucrose diet (HSD) or a high-fat plus high-sucrose diet (HFSD) for 6 months. Eyes were examined using multimodal imaging modalities and electroretinograms. Retinal sections were analyzed using H&E staining, Toluidine Blue staining, immunostaining and transmission electron microscopy. Lipids and complement C3 protein (C3) in serum or aqueous humor were measured. RNA sequencing was performed to evaluate the retinal transcriptomes. HFD and HSD had minor effects on lipid profiles but, when fed concomitantly, synergistically induced severe dyslipidemia. None of the four diets caused obesity. HFSD induced retinal lesions, such as reticular pseudodrusen (RPDs) and other pigmentary abnormalities. RPD-like lesions were mainly lipid droplets around cells of the retinal pigment epithelium. HFSD also induced elevated levels of ocular C3 and reduced the density of retinal vessels. In conclusion, HFD and HSD can – when combined − induce normal-weight dyslipidemia and RPD-like retinal lesions. HFSD-fed male Chinchilla rabbits are a good model of early AMD.

## Teaser

**Summary:** High-fat and high-sucrose diets for 6 months can synergistically induce severe dyslipidemia and RPD-like retinal lesions (mainly lipid droplets around RPE cells) without obesity in male Chinchilla rabbits.

## INTRODUCTION

Age-related macular degeneration (AMD) is a leading cause of vision loss in older people, accounting for 6–9% of legal blindness globally (Fleckenstein et al., 2021; Jonas et al., 2017). AMD affects photoreceptors, Müller cells, retinal pigment epithelium (RPE), Bruch's membrane (BrM), choriocapillaris structures and deep capillary plexus networks in the retinal vasculature, mainly in the macular region (Chen et al., 2022). Clinically, AMD can be divided into early, intermediate and late (advanced) stages (Ferris et al., 2013). At early stage, AMD is characterized by accumulating medium-sized (63-125 µm) extracellular deposits (drusen). At intermediate stage, drusen become large and more confluent, and RPE cells degenerate. Late stage ADM can be either geographic atrophy, i.e. dry AMD, or neovascular AMD (wet AMD). Loss of photoreceptors, RPE and choriocapillaris is definitive in dry AMD, and invasion of macular neovascularization (MNV) into the outer retina, subretinal space or sub-RPE space is the feature of wet AMD (Ferris et al., 2013; Fleckenstein et al., 2021).

While intravitreal injections of anti-vascular endothelial growth factor (anti-VEGF) drugs have greatly improved the visual outcome of wet AMD (Brynskov et al., 2020), there is no treatment for dry AMD, which affects most patients. Progression from earlier AMD stages to dry AMD can be partially delayed by diets high in omega-3 long-chain polyunsaturated fatty acids and carotenoids (lutein, zeaxanthin, and beta-carotene), as diet is one of the most important environmental risk factors of AMD (Chew et al., 2013; Rozing et al., 2020). High intakes of cholesterol and saturated fats have long been considered critical in AMD development (van Leeuwen et al., 2018).

Drusen are focal extracellular deposits located between the basal lamina of the RPE and the inner collagenous layer of the BrM, i.e. the sub-RPE-BL space (Li et al., 2018). Reticular pseudodrusen (RPDs; also known as subretinal drusenoid deposits), are a different type of deposit present in the subretinal space on the apical side of the RPE in AMD patients (Wu et al., 2022). Unlike conventional soft drusen, which are predominately under the cone-rich fovea, RPDs are more likely to appear in perifoveal regions with a high density of rods. RPDs are a critical phenotype contributing to AMD progression, and correlate with an increased risk of dry AMD and MNV development (Duic et al., 2024).

Several studies have indicated a pathogenic role of altered lipid metabolism in AMD development (Lin et al., 2022). First, drusen are mainly (∼40%) composed of lipids protein and minerals (Curcio, 2018; Wang et al., 2010). RPDs also have lots of lysolipids (LysoPCs, LysoPEs and LysoPAs) (Anderson et al., 2023). It has been proposed that formation of drusen is initiated by lipid droplets in the sub-RPE-BL space, followed by mineralization to form tiny hydroxyapatite (HAP) spherules and binding of protein to HAP surfaces (Bergen et al., 2019; Thompson et al., 2015). Lipid droplets might also affect the formation of RPDs, as the secretory phospholipase A2 family member (PLA2G5) has been linked to formation of both lipid droplets and RPDs (Anderson et al., 2023; Guijas et al., 2014). Second, genome-wide association studies have indicated that polymorphisms within or near the loci of several genes − such as *ABCA1*, *APOE*, *CETP* and *LIPC* genes, whose protein products are involved in lipid metabolism − are associated with AMD (Acar et al., 2020; Fritsche et al., 2016; Han et al., 2020). Third, some studies have found that increased levels of serum high-density lipoprotein (HDL) are associated with increased risk of AMD, whereas elevated serum levels of total cholesterol (TC), low-density lipoprotein (LDL) and triglycerides (TGs) are associated with a decreased risk of AMD (Colijn et al., 2019; Sasaki et al., 2018; Wang et al., 2016). Based on these findings, it has been proposed that HDL is a potential therapeutic target for AMD (Kelly et al., 2020). These findings challenge the paradigm that LDL is ‘bad’ cholesterol and HDL is ‘good’ cholesterol (Lin et al., 2022). However, there has also been an association between early AMD and elevated total cholesterol and LDL levels in female participants (Sasaki et al., 2018). Some recent studies have reported no significant associations between serum lipoprotein profiles and AMD (Lüdtke et al., 2019; Mao et al., 2019). Whether systemic lipid profiles directly influence AMD development or represent lipid metabolism in the retina is unknown and deserves further investigation.

In addition to dyslipidemia, hyperglycemia or diabetes are also a risk factor for AMD (Choi et al., 2011; He et al., 2018). The underlying mechanisms might be related to functional and structural changes in the RPE, BrM, or choroidal circulation in diabetic patients; for instance, hyperglycemia-induced chronic inflammation and the accumulation of advanced glycation end products (AGE) may contribute alterations in the choriocapillaris basement membrane and BrM (Hwang et al., 2023; Wang et al., 2017).

Diet-induced animal models are a powerful tool for understanding how lipids and sugar are involved in AMD development. Commonly used animal models are rodents and rabbits. Many studies have used rodents as the model system; for instance, consumption of a high-glycemia (HG) diet but not a low-glycemia (LG) diet induces many AMD features (Andriessen et al., 2016; Rowan et al., 2017). Switching from HG to LG diet late in life can reverse these AMD features, implying the critical role glucose has in development of AMD (Rowan et al., 2017). An HFD (60% kcal fat) can exacerbate the laser–induced photocoagulation model of choroidal neovascularization in mice (Andriessen et al., 2016).

Rabbits are also a useful model because they represent an intermediate between large (pigs, dogs, monkeys) and small (rodents) animal models (Lozano et al., 2019). Rabbits are the most common animal model used for preclinical safety evaluation of ocular drugs, as eye anatomy is similar in humans and rabbits, and because rabbits are meek and easy to handle (Del Amo and Urtti, 2015). As herbivorous animals, rabbits are susceptible to HFDs, and, like humans, have high baseline profiles of the plasma lipid transfer protein cholesteryl ester transfer protein (CETP) and of LDL (Fan et al., 2015). A cholesterol-enriched diet for 3 months induces astrogliosis, drusen-like deposits, and cholesterol accumulations in the retinas of male New Zealand white rabbits (Dasari et al., 2011), and these AMD features can be rescued by low doses of statins (Fernandez-Navarro et al., 2016).

Feeding a HFD, high-sucrose diet (HSD) or high-fat plus high-sucrose diet (HFSD) has also been used in rabbit models to trigger obesity and metabolic syndromes, including hyperglycemia, insulin resistance, dyslipidemia and increased levels of free fatty acids in the blood (Lozano et al., 2019). Still, retinal phenotypes of these rabbits have not yet been systemically analyzed (Helfenstein et al., 2011). Male rabbits tend to be resistant to obesity under these diets (Waqar et al., 2010), and pigmented rabbits have a different electroretinogram (ERG) pattern compared to that of albino rabbits, such as New Zealand rabbits (Ioshimoto et al., 2018). Thus, our study compared the retinal phenotypes of pigmented male Chinchilla rabbits in response to HFD, HSD or HFSD over 6 months.

## RESULTS

### HFD and HSD synergistically induce dyslipidemia but do not cause obesity in male Chinchilla rabbits

**Fig. 1.**
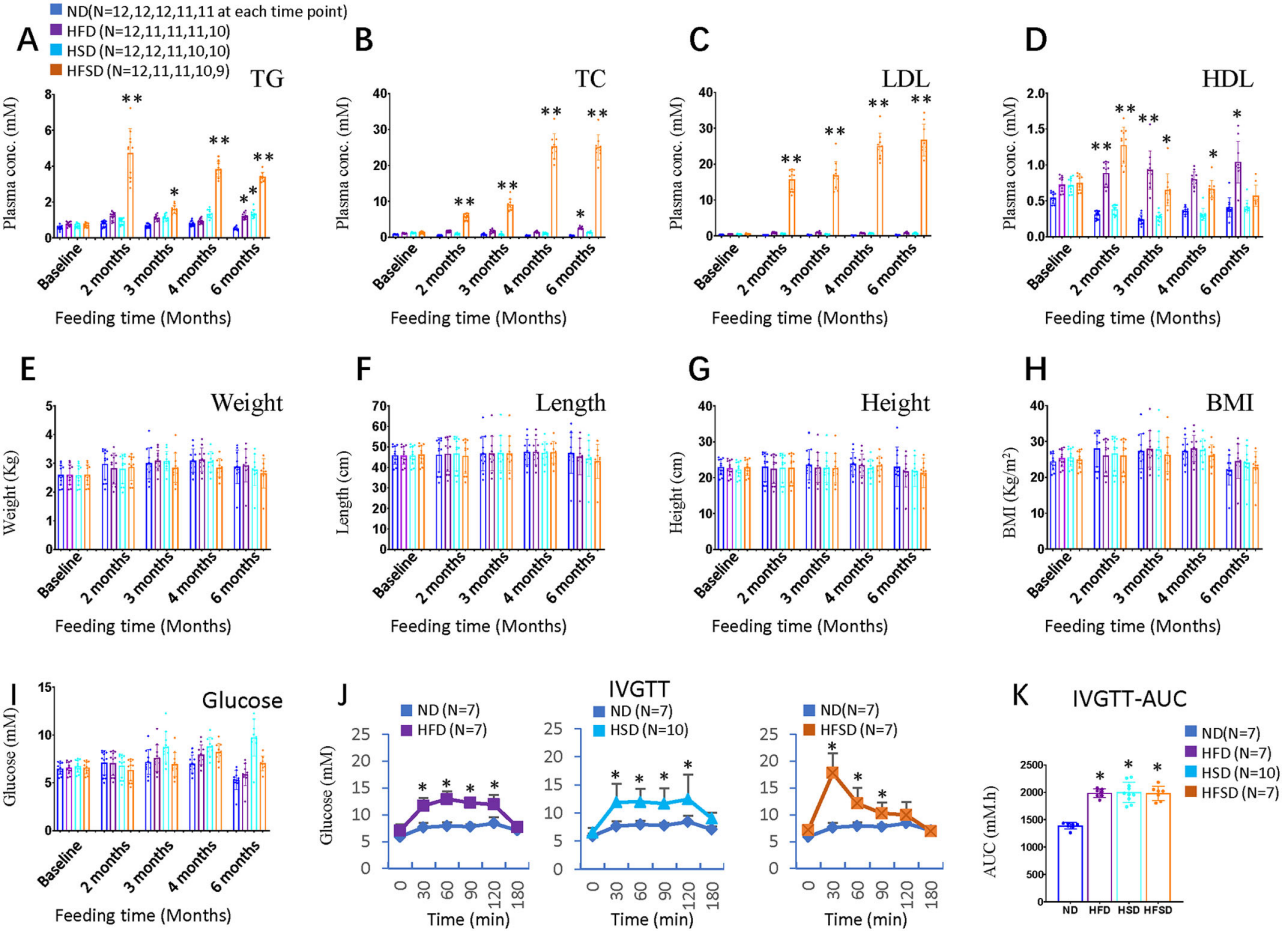
**Comparison of plasma lipid profiles, body mass index (BMI) and glucose metabolism among indicated diet groups and feeding times as indicated.** (A) triglycerides (TG), (B) total cholesterol (TC), (C) low-density lipoprotein (LDL), (D) high-density lipoprotein (HDL), (E) weight, (F) length, (G) height, (H) BMI, (I) random plasma glucose, (J) Intravenous glucose tolerance test (IVGTT) test, (K) IVGTT-area under the curve (AUC) analysis. Error bars represent the ±s.d. Significance was determined using one-way ANOVA followed by Bonferroni correction (*P<0.05, **P<0.01). ND, normal diet; HFD, high-fat diet; HSD, high-sucrose diet; HFSD, high-fat plus high-sucrose diet.

In general, rabbits are susceptible to a high-cholesterol diet and can rapidly develop severe hypercholesterolemia (Fan et al., 2015). A HFSD over 3 months can induce weight gain in male Japanese white rabbits (Zhao et al., 2008). However, we found that a HFD (10% lard, 0.5% cholesterol) or HSD (40% sucrose) for 6 months only had minor effects on blood triglycerides, total cholesterol and LDL cholesterol profiles in male Chinchilla rabbits (Fig. 1A-C). HFD only slightly increased triglyceride and total cholesterol levels at the end of the experiment, and HSD only increased triglyceride levels at the end. They had no effects on levels of LDL cholesterol throughout the experiment (Fig. 1A-C). HFD significantly increased HDL levels, while HSD did not affect HDL throughout the experiment (Fig. 1D). As such, they did not affect weight, length, height and BMI of the rabbits throughout the experiment, and no obesity occurred in them (Fig. 1E-H). Even though HFD did not induce high serum levels of triglyceride or total cholesterol during the experiment, it caused substantial liver fibrosis (Fig. S1), suggesting it induces fat liver, as liver fibrosis is a common outcome of steatosis (Farrell and Larter, 2006).

Surprisingly, a HFSD (10% lard, 0.5% cholesterol, 35% sucrose) significantly increased levels of triglyceride, total cholesterol and LDL (Fig. 1A-C), indicating that concomitant high intake of fat and sucrose has synergistic effects on these lipids. HFSD as well as HFD had a similar impact on levels of HDL levels, i.e. both increased its levels (Fig. 1D). However, even though HFSD increased levels of triglycerides, total cholesterol and LDL, it did not induce obesity or affect weight, length, height or BMI throughout the experiment (Fig. 1E-H).

### HFD and HSD have similar but not synergistic effects on glucose metabolism

Blood glucose levels were comparable between all groups throughout the experiment; i.e. slightly elevated plasma glucose levels were noticed in HSD and HFSD groups at the end of the experiment (Fig. 1I). Intravenous glucose tolerance test (IVGTT) was performed at the end of the experiment. Generally, IVGTT results were comparable between experimental groups, i.e. all three groups showed a slower rate of glucose clearance (Fig. 1J). Correspondingly, the area under the curve (AUC) calculated using the IVGTT results increased in all three groups similarly (Fig. 1K). These results indicate that both HFD and HSD damaged glucose tolerance but did not have any synergistic effects on glucose metabolism.

### HFD and HSD synergistically induce abnormal ERG

Standard International Society for Clinical Electrophysiology of Vision (ISCEV) guidelines regarding ERG protocols suggest three scotopic ERG and two photopic ERG tests (Robson et al., 2022). The dark-adapted scotopic ERGs include responses to flash strengths of 0.01 and 3 phot cd·s·m^−2^ (scotopic 0.01 ERG and scotopic 3.0 ERG and scotopic 3.0 OPs). The scotopic 0.01 ERG measures the response of the rod bipolar cells and is dependent on input from rod photoreceptors. Scotopic 0.01 ERGs only have b-waves. Scotopic 3.0 ERGs are mixed rod and cone system responses, with the rod system contribution dominating in a healthy retina. Both of them have a- and b-waves, the former representing rod photoreceptor function and the latter rod-driven On-bipolar cells. Oscillatory potentials (OPs) are relatively high-frequency and low-amplitude components on the rising limb of the scotopic 3.0 ERGs b-waves (Scotopic 3.0 OPs), which reflect inner retinal activity involving amacrine cells and retinal ganglion cells.

**Fig. 2.**
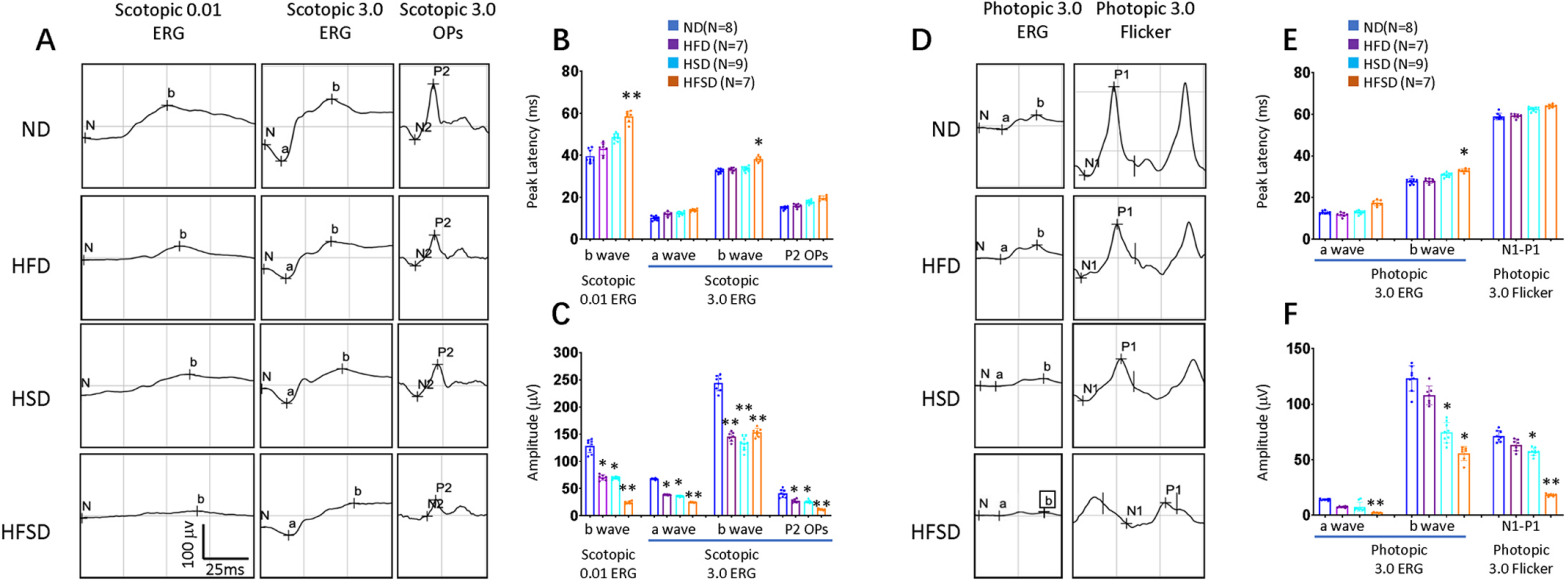
**Full-field flash ERG changes in eyes obtained from rabbits after feeding an ND (control), HFD, HSD or HSFD for 6 months.** (A) Representative scotopic ERG recordings according to ISCEV guidelines (three types of measurement as indicated). (B) Quantification of peak latency of indicated scotopic ERG waves (a-, b-, P2-wave) of scotopic OPs and scotopic groups as indicated. (C) Quantification of the amplitude of indicated scotopic ERG waves (a-, b-, P2-wave) of scotopic Ops as indicated. (D) Representative photopic ERG recordings according to ISCEV guidelines (two types of measurement) as indicated. (E) Quantification of peak latency of indicated photopic ERG waves (a-, b-, N1-P1-wave) of photopic flicker 30 Hz as indicated. (F) Quantification of the amplitude of photopic ERG waves (a-, b-, N1-P1-wave) of photopic flicker 30 Hz as indicated. Error bars represent ±s.d. Significance was determined using one-way ANOVA followed by Bonferroni correction (*P<0.05, **P<0.01). ND, normal diet; HFD, high-fat diet; HSD, high-sucrose diet; HFSD, high-fat plus high-sucrose diet.

After 6 months of feeding, animals from the HFD and HSD groups showed no changes in peak latency time of the waves (a-, b- and P2-waves of OPs) of the scotopic ERGs (Fig. 2A,B), while animals from the HFSD group showed prolonged peak latency of scotopic b-waves (Fig. 2A,B). Still, HFD and HSD-fed animals had a minor reduction of amplitude in all waves of the scotopic ERGs (Fig. 2A,C). At the same time, HFSD-fed animals showed a more considerable amplitude reduction in all waves of the scotopic ERGs (Fig. 2A,C), suggesting HFD and HSD act synergistically on inhibiting the retinal function of the rod system.

The light-adapted photopic tests included two types of photopic ERG, recorded to provide different but complementary measures of cone system function (Robson et al., 2022). Photopic 3.0 ERGs (single flash) have both a- and b-waves. The a-wave represents the activity of the Off-bipolar cells. The b-wave represents On- and Off-bipolar cell activity, with contributions mediated by cone mechanisms. Photopic 3.0 Flicker (30 Hz) ERG is generated mainly by cone On- and Off- bipolar cells but also depends on the cone functions (Robson et al., 2022).

After 6 months of feeding, animals from the HFD and HSD groups showed no changes in peak latency time of the photopic waves of the ERGs (a-, b- and N1-P1-waves of 30 Hz flickers) (Fig. 2D,E), while animals from the HFSD group showed prolonged peak latency of the photopic a waves (Fig. 2D,E). The HFD also had no effect on the amplitude of either wave of the photopic ERGs (Fig. 2D), suggesting it does not impact cone system in rabbit. Interestingly, HSD had a minor amplitude reduction in b- and N1-P1-waves of the photopic ERGs (Fig. 2D,F). At the same time, HFSD showed a more considerable amplitude reduction in all waves of the photopic ERGs (Fig. 2D,F), suggesting that HFD and HSD act synergistically on inhibiting the retinal function of the cone system.

### HFSD induces reticular pseudodrusen-like lesions and pigmentary abnormalities

**Fig. 3.**
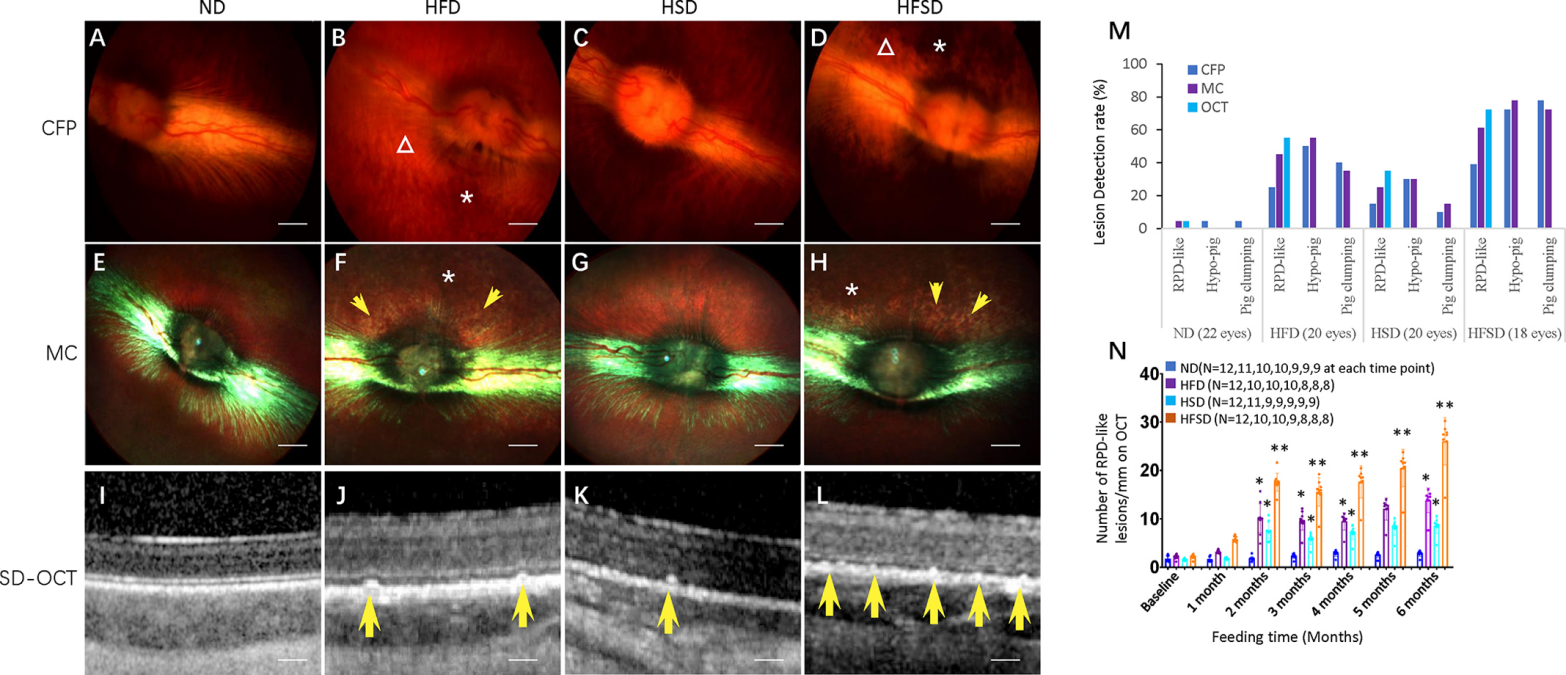
**Color fundus photographs (CFPs), multicolor (MC) and SD-OCT images of rabbit eyes.** (A-D) CFPs, taken by a TRC-50DX retinal camera. (E-L) MC (E-H) and SD-OCT (I-L) images, taken by the Heidelberg multimodal imaging system Spectralis. CFPs of eyes obtained from HFD- and HFSD-fed rabbits show saffron yellow areas of hypopigmentation (see triangle in B and D) and brown-black areas of pigment clumping (see asterisk in B and D). In MC images, the latter appeared grey and brown (see asterisk in F and H). MC images also show RPD-like lesions as lime flecks adjacent to the optic disk and vascular arcades in HFD- and HFSD-fed rabbits (see yellow arrows in F and H). Punctate RPD-like lesions (see yellow arrows in I-L) were mainly found in HFD-, HSD- and HFSD-fed rabbits. (M) Lesion detection rates (in %) at the end of the experiment, i.e. after 6 months of feeding diets as indicated, established by using imaging techniques (CFP, MC and OCT) as indicated. (N) Quantification of RPD-like lesions (per mm-length) of the outer retina as shown in I-L. Error bars represent ±s.d. Significance was determined using one-way ANOVA followed by Bonferroni correction (*P<0.05, **P<0.01). RPD-like, RPD-like deposits; Hypo-pig, hypopigmentation; Pig clumping, pigmentation clumping. ND, normal diet; HFD, high-fat diet; HSD, high-sucrose diet; HFSD, high-fat plus high-sucrose diet. Scale bars: 200 μm.

We followed the ocular fundus changes during monthly retinal examinations by using MultiColour (MC) fundus photography (Wang et al., 2021), colour fundus photography (CFP) (Wang et al., 2021) and optical coherence tomography (OCT) (Wang et al., 2021), and found three significant retinal changes that included reticular pseudodrusen-like (RPD-like) lesions, hypopigmentation and pigment clumping (Fig. 3). RPD-like lesions appeared as yellowish dots or lime flecks, often in areas adjacent to the optic disk or vascular arcades within the mid retina, resembling RPDs of AMD patients (Fig. 3A-H). In spectral domain-OCT (SD-OCT) images, RPD-like lesions appeared as high-density dome-shaped spots located between the RPE and the ellipsoid zone (EZ) (Fig. 3I-L).

These RPD-like lesions observed in the HFSD-fed group appeared more visible on MC (61.1%) and SD-OCT (72.2%) compared with CFP (38.9%) (Fig. 3M). Eyes of HFSD- or HFD-fed rabbits also showed more RPD-like lesions in MC and OCT images compared with rabbits fed an ND or HSD (Fig. 3M). These RPD-like lesions form during the second month of feeding a HFD, HSD or HFSD, and increased with feeding duration, especially in the HFD and HFSD groups (Fig. 3N).

Hypopigmentation was seen as saffron-yellow areas in CFP images, and grey and yellow in MC images (Fig. 3) – similar to hypopigmentation in AMD patients. Pigment-clumping areas were dark brown in CFP images (Fig. 3B,D), and grey and brown in MC images (Fig. 3F,H). Both pigment changes were more frequently detected in HFSD and HFD groups than in the ND group (Fig. 3M). In addition, both hypopigmentation and pigment-clumping areas had similar detection frequencies with CFP and MC imaging. They were more frequently detected in the HFSD and HFD groups compared with the ND group (Fig. 3M). However, as OCT cannot detect retinal pigment changes (Fig. 3M), monitoring these lesions was very challenging during the experiment.

### HFSD induces lipid droplets around RPE cells and retinal degeneration

**Fig. 4.**
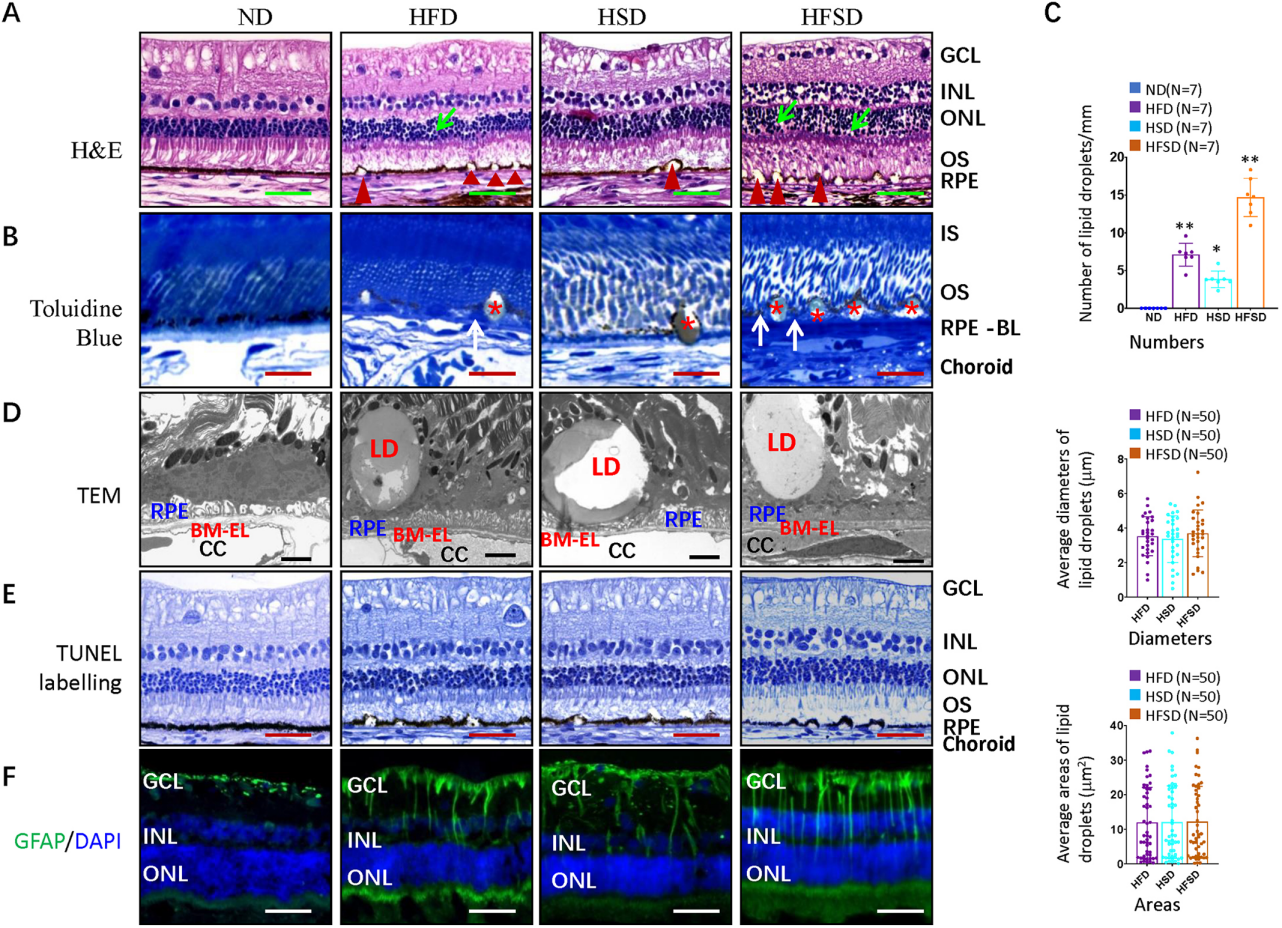
**Light, transmission electron and fluorescence microscopy images showing stained rabbit retinas.** (A-F) Retinas of rabbits that had been fed different diets for 6 months as indicated were analyzed, using H&E staining (A), Toluidine Blue staining (B), TEM (D), TUNEL labeling (E) or staining for GFAP (F). H&E staining shows retinal pyknotic nuclei (green arrows), lipid droplets between RPE and rod outer segments (red arrowheads). (B) Toluidine Blue staining shows lipid deposits (asterisks) and mound as basal laminar deposits (BlamD, white arrows) internal to the RPE basement membrane. (C) Quantification of the number of lipid droplets (per mm length of the outer retina (top), the average diameter of lipid droplets (in μm), and the average area of lipid droplets (in μm^2^) in retinas of diet groups as indicated based on H&E staining. (D) TEM images of retinal pigment epithelium (RPE) cells and Bruch's membrane show lipid droplets (LD), RPE cells, an elastic layer of Bruch's membrane (BM-EL) and choriocapillaris (CC) structures. (E) TUNEL labeling of retinal sections obtained from diet groups as indicated stained for nuclei (hematoxylin, blue) and cell death (TUNEL, brown). (F) Retinal sections from diet groups as indicated stained for nuclei (DAPI, blue) and GFAP (green). Error bars represent ±s.d. Significance was determined using one-way ANOVA followed by Bonferroni correction (*P<0.05, **P<0.01). RPE-BL, basal laminar of retinal pigment epithelium; IS, photoreceptor inner segments; OS, photoreceptor outer segments; ONL, outer nuclear layer; INL, inner nuclear layer; GCL, ganglion cell layer. ND, normal diet; HFD, high-fat diet; HSD, high-sucrose diet; HFSD, high-fat plus high-sucrose diet. Scale bars: 100 μm (A,E,F), 20 μm (C), 1 μm (D).

The most striking feature when using hematoxylin and eosin (H&E) staining of the retina obtained from our experimental rabbits was the occurrence of dome-shaped vacuoles between the RPE-BrM complex and the outer segment of photoreceptors (Fig. 4A), which were confirmed by Toluidine Blue staining of semi-thin sections (Fig. 4B). In contrast to SD-OCT imaging, which identified very few RPD-like lesions in the ND-fed group (Fig. 3N), we did not observe these lesions in ND-fed animals when using H&E staining.

Consistent with SD-OCT results (Fig. 3N), the number of lipid droplets around RPE was highest in the HFSD-fed group, followed by HFD- and HSD-fed groups (Fig. 4C). However, size and area of these lipid droplets varied, and we found no significant differences between these groups (Fig. 4C). Transmission electron microscopy (TEM) also revealed that the dome-shaped vacuoles were lipid droplets surrounding RPE cells in the subretinal space (Fig. 4D). We also observed other pathological retinal changes. First, staining with Toluidine Blue identified basal laminar deposits (BlamD) that accumulate internally to the RPE basement membrane in HFD and HFSD eyes (Fig. 4B). Second, there were some features of retinal degeneration, such as pyknotic nuclei in the ONL (Fig. 4A). However, TUNEL staining at this stage failed to identify any cell death (Fig. 4E). As TUNEL staining primarily detects apoptosis, other types of cell death, such as necrosis and pyroptosis, may not be detected by TUNEL staining and, therefore, may be presented in this animal model. However, staining for glial fibrillary acidic protein (GFAP) to identify activated Müller glia, suggested reactive gliosis in the three experimental groups (Fig. 4F), supporting the notion of retinal degeneration.

### HFSD increased the C3 level in the eye

**Fig. 5.**
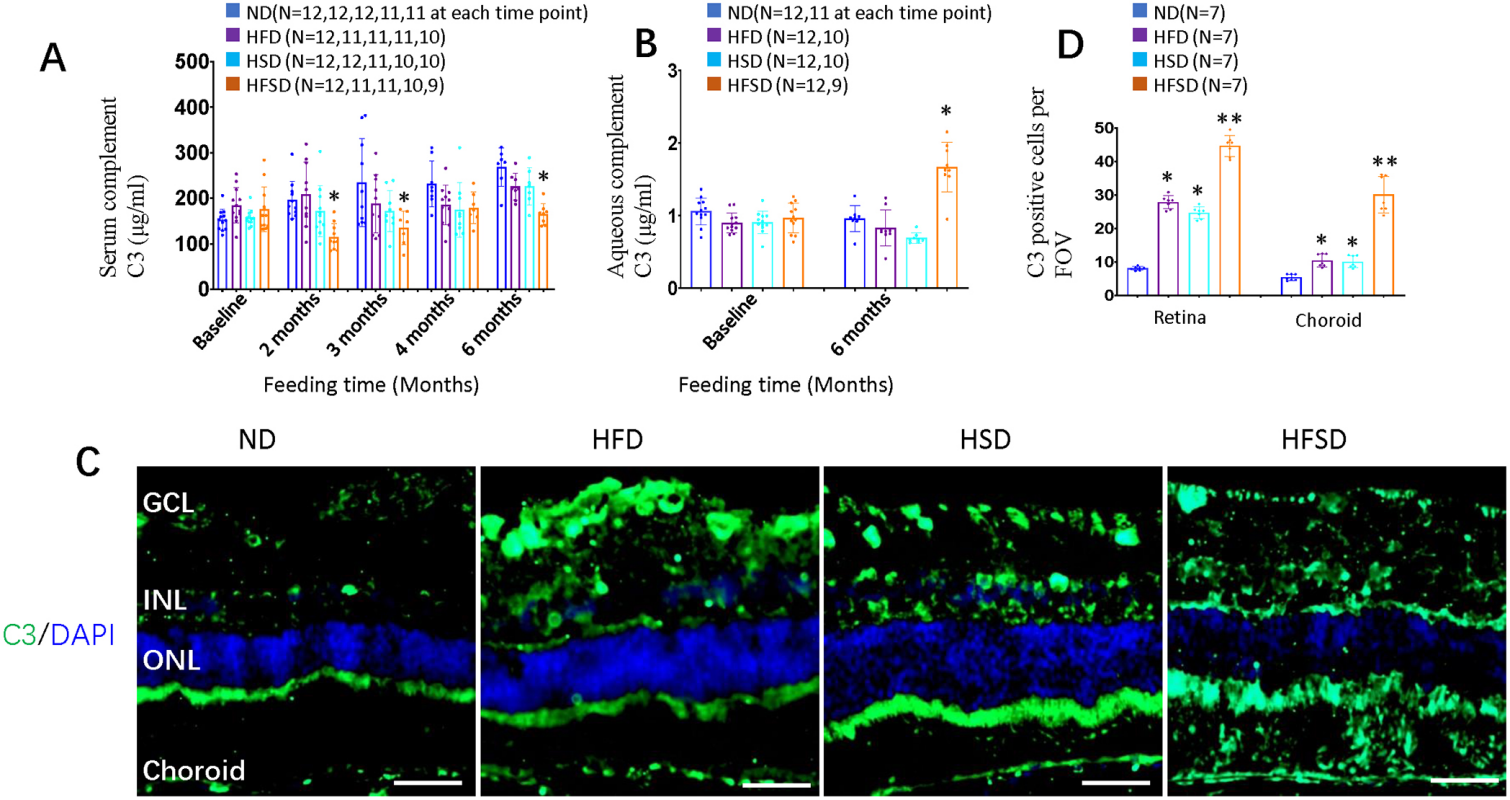
**Effects of ND (control), HFD, HSD or HSFD on levels of C3 in the peripheral blood plasma and aqueous humor of the eye.** (A) Serum concentration (in µg/ml) of complement C3 at time points or in diet groups as indicated, showing that a HSFD results in reduced levels of C3. (B) Aqueous humor concentration (in µg/ml) of complement C3 at time points or in diet groups as indicated, showing that, after 6 months, a HFSD results in increased levels of ocular C3. (C) Horizontal retinal sections of indicated groups after 6 months of feeding were stained for nucleus (DAPI, blue) and C3 (green). ONL: outer nuclear layer; INL: inner nuclear layer; GCL: ganglion cell layer. Scale bars: 50 μm. (D) Quantification of C3-positive cells per field of view (FOV) in the retina and choroid in diet groups as indicated, showing that HFD, HSD or HFSD all increased the number of C3-positive cells. Error bars represent ±s.d. Significance was determined using one-way ANOVA followed by Bonferroni correction (*P<0.05, **P<0.01). ND, normal diet; HFD, high-fat diet; HSD, high-sucrose diet; HFSD, high-fat plus high-sucrose diet. Scale bars: 100 mm.

The level of circulating C3 was followed monthly, and aqueous C3 was measured at the start and end of the experiment (Fig. 5A,B). At the end of the experiment, C3 levels of the HFSD group were lower in the serum (Fig. 5A) but higher in the aqueous humor compared with the control group (Fig. 5B). The increase of C3 in the eye was confirmed by immunological staining of C3 in the retina (Fig. 5C,D).

### HFSD reduced retinal vessel density

**Fig. 6.**
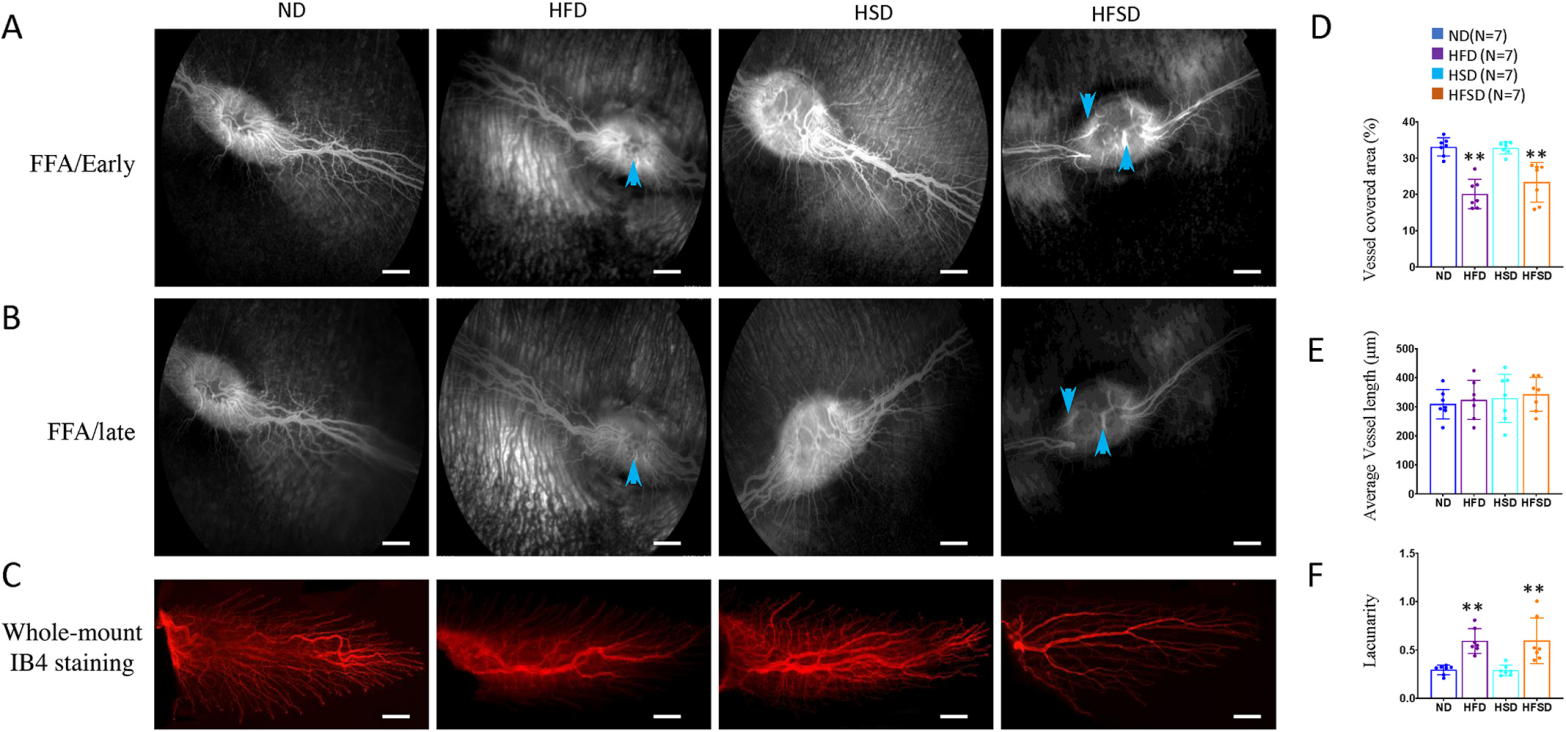
**Changes in retinal vasculature and circulation in rabbits after feeding an ND (control), HFD, HSD or HSFD for 6 months.** (A,B) Fundus fluorescein angiograph (FFA) images (early phases, A; late phases, B) were taken by the Heidelberg multimodal imaging system Spectralis. Blue arrows indicate fluorescence filling defects, suggesting interruption of blood flow. (C) Whole-mount retinal staining for IB4 (red) to detect retinal vessels in rabbits after 6 months of feeding as indicated. (D-F) Quantification of vessel-covered area (in %) (D), average vessel length (in μm) (E) and lacunarity (F) after whole-mount IB4 staining for microglia in rabbit retinas as indicated. Error bars represent ±s.d. Significance was determined using one-way ANOVA followed by Bonferroni correction (**P<0.01). ND, normal diet; HFD, high-fat diet; HSD, high-sucrose diet; HFSD, high-fat plus high-sucrose diet. Scale bars: 50 µm.

Fundus fluorescein angiography (FFA) showed a merangiotic retinal vascular pattern in rabbits. During the early and late phases of FFA, HFD and HFSD rabbits had much fewer fluorescent labelled retinal vessel branches than control rabbits (Fig. 6A,B). The fluorescent labelling of retinal vessels was not continuous in the HFD and HFSD rabbits, suggesting fluorescent labelling defects and interruption of blood flows (Fig. 6A,B, blue arrows). The retinal vascular abnormalities were further examined on whole-mount preparations stained for vascular endothelial cells by using isolectin B_4_ (IB4), which confirmed the vascular density reduction of retinal vessels (reduced vessel area, increased lacunarity), while the average vessel length had not changed (Fig. 6C-F). HSDs did not affect retinal blood vessels (Fig. 6C-F).

### RNA sequencing revealed changes in the metabolism of the rabbit retina

To understand the mechanisms of the above retinal phenotypes, we performed RNA sequencing of retinal cells (two retinas examined per group) obtained from ND-, HFD-, HSD- or HFSD-fed rabbits after 6 months of feeding.

We identified 189, 523 or 433 differentially expressed genes (DEGs) in retinas from rabbits on HFD, HSD or HFSD, respectively, compared to ND retinas (Table S1 and GSE276438 in GEO database).

Gene Ontology (GO) analysis indicated that the most upregulated GO biological process included Muscle Contraction (GO: 0006936), Sarcoplasmic Reticulum Calcium Ion Transport (GO: 0070296) and Negative Regulation of VEGF Signaling Pathway (GO: 1900747), while most downregulated GO biological process included Blood-Brain Barrier (GO: 0150104), Vascular Transport (GO: 0010232), Regulation of Angiogenesis (GO: 0045765), Vitamin A Metabolic Process (GO: 0006776), Retinoid Metabolic Process (GO: 0001523), Melanin Biosynthetic Process (GO: 0042438), Sensory Perception of Light (GO: 0050953) and TGF Beta Receptor Pathway (GO: 0007179) (Fig. S2A,B).

**Fig. 7.**
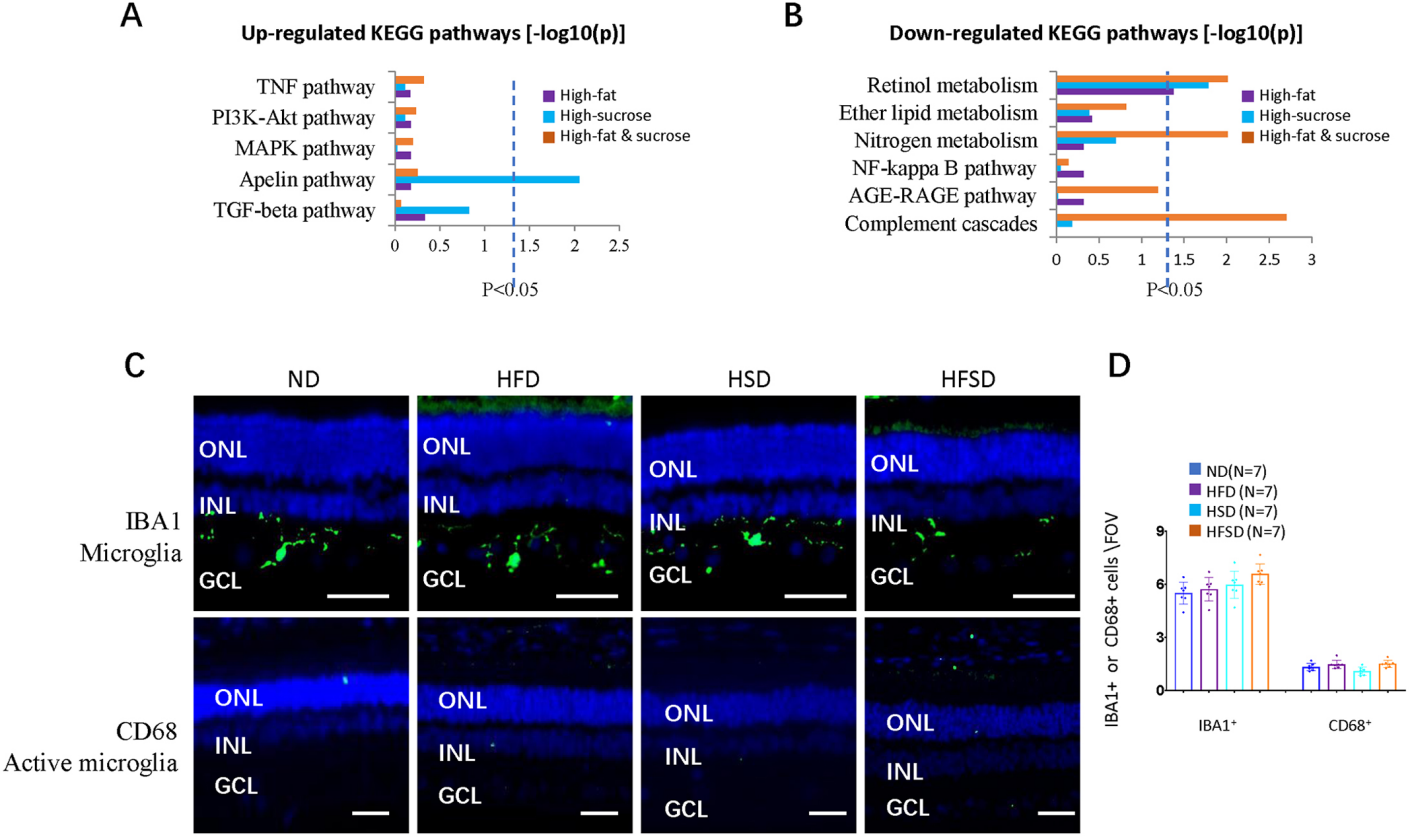
**Retinal RNA sequencing, IBA1^+^ and CD68^+^ microglia in rabbits after feeding an ND (control), HFD, HSD or HSFD for 6 months.** (A,B) Gene list enrichment analysis using Kyoto Encyclopedia of Genes (KEGG) 2022 data sets in Enrichr of upregulated DEGs (A) and downregulated DEGs (B) (−log10 P). Dotted line indicates *P*<0.05. (C) Horizontal retinal sections in rabbits after 6 months of feeding as indicated, showing nuclei (DAPI, blue), retinal microglia (IBA1, green) and activated microglia (CD68, green). ONL: outer nuclear layer; INL: inner nuclear layer; GCL: ganglion cell layer. Scale bars: 50 µm. (D) Quantification of IBA1-positive (IBA1^+^) or CD68-positive (CD68^+^) cells per field of view (FOV) in retinas of rabbits after 6 months of feeding as indicated. Error bars represent the ±s.d. of measurements from at least seven animals. ND, normal diet; HFD, high-fat diet; HSD, high-sucrose diet; HFSD, high-fat plus high-sucrose diet.

Gene list enrichment analysis using Enrichr (https://maayanlab.cloud/Enrichr/) indicated only one enriched pathway (Apelin pathway) of upregulated DEGs, present in the HSD group only (Fig. 7A). The Apelin pathway is mainly related to energy metabolism and, possibly, some inflammatory pathways (Hu et al., 2021). However, common inflammatory pathways, including those of TNF, PI3K−Akt, MAPK and TGF-β, were not enriched in upregulated DEGs (Fig. 7A). GO term analysis suggested downregulation of molecular functions of MAP Kinase Tyrosine Phosphatase Activity (GO: 0033550) and Type II TGF Beta Receptor Binding (GO: 0005114) (Fig. S2F).

For the downregulated DEGs, retinol metabolism genes (including *CYP26B1*, *CYP26C1*, *RDH5*, *RDH10*, *ALDH1A1*, *LRAT*, *BCO1* and *RPE65*) were downregulated in all three groups (Fig. 7B), which is consistent with findings that they all form lipid droplets around RPE cells. Nitrogen metabolism genes were also downregulated in the HFSD group (Fig. 7B). Interestingly, while we found C3 protein expression to be upregulated in the intraocular fluid of the HFSD group (Fig. 5B,D), genes of the complement system (including *SERPINB2*, *SERPING1*, *C1S*, *C1R*, *C1QC*, *F2R*, *F3* and *BDKRB2*) were downregulated in retinas of the HFSD group (Fig. 7B). As inflammation is closely related to AMD development, we used staining for the retinal microglial cell marker IBA1 and the microglia activation marker CD68. We found no differences between control and HFD, HSD, HFSD groups for either marker (Fig. 7C,D).

## DISCUSSION

In this study, we evaluated the long-term effect HFD, HSD or HFSD has on the retina of male Chinchilla rabbits. We found that male Chinchilla rabbits on HFSD are a potential animal model to study early AMD and normal-weight dyslipidemia (NWD), and that these phenotypes depend on their synergistic effects on lipid metabolism, including retinol metabolism.

### The advantages of rabbits as an AMD model

To establish a translatable model of dry AMD is a great challenge. Several key factors, such as animal species, experimental inducer and analytic methods, can influence the model's success. For animal species, the eye size is our first consideration. Eyes of appropriate size allows us to perform *in vivo* ophthalmic examinations stably and follow the development of dry AMD-like fundus changes. Rodents with tiny eyeballs and large lenses hinder the application of ophthalmic instruments. They limit the quality of fundus images, therefore, introducing unsatisfying *in vivo* ophthalmic results in most rodent studies (Clarkson-Townsend et al., 2021). Non-human primates have the appropriate eye size and even macula structure but cost and availability challenge their usability as an efficient model. As an affordable animal species, rabbits have the appropriate eye size and have been used for various ophthalmic drug tests (Ramirez et al., 2006). The possibility of using clinical imaging systems to monitor their fundus changes made us choose the chinchilla-pigmented rabbit as our model species. As rabbits have no macular area and a melanotic vascular pattern, we here used the optic disc and surrounding area as the area of interest.

### Synergistic effects of fat and sucrose on lipid metabolism

In our study, feeding a HFD (10% lard, 0.5% cholesterol) or HSD (40% sucrose) for 6 months only had minor effects on total cholesterol, triglycerides and LDL. Female New Zealand white rabbits were fed a diet of 10% corn oil and 5% lard, which increased the serum triglycerides but not total cholesterol (Carroll et al., 1996). Male Japanese white rabbits fed with 10% coconut oil over 5 months did not show effects on triglyceride, total cholesterol, HDL or glucose serum levels (Waqar et al., 2010). Thus, the effects of HFDs on rabbits are gender- and breed-specific. Male New Zealand white rabbits fed a diet of 4% peanut oil and 0.5% cholesterol over 3 months showed increased levels of serum triglyceride and total cholesterol, supporting the idea that rabbits are susceptible to cholesterol-enriched diets (Filippi et al., 2009; Marchiani et al., 2015). Based on our observation that a HFD (0.5% cholesterol) in combination with normal sucrose intake did not induce hypercholesterolemia, we concluded that the Chinchilla rabbit breed used in our experiments is somehow resistant to the negative effects of a high-cholesterol diet. However, a HFSD (10% lard, 0.5% cholesterol, 35% sucrose) induced severe dyslipidemia, hypercholesterolemia and high levels of serum LDL, suggesting fat and sucrose act synergistically to induce dyslipidemia in Chinchilla rabbits. This has been observed in a mouse model of fatty liver and depends on the critical enzyme fructokinase ketohexokinase (KHK) in fructose metabolism (Ishimoto et al., 2013). In brief, HFD could only induce steatosis, while HFSD induced more-severe steatosis with other complications. Despite similar energy intake, KHK-null mice fed an HFSD were protected from developing hepatic inflammation and fibrosis. The mechanism for fructose-induced fatty liver is dependent on KHK isoform C (KHK-C) that is distinct from most nutrition-based enzymes since it causes transient ATP depletion before the downstream production of ATP (Ishimoto et al., 2012). If this enzyme is also the central mechanism of this synergistic effect in Chinchilla rabbits, it deserves further investigation. In the future, HFSD plus KHK-C delivery may enhance the phenotypes of this animal model (Herman and Birnbaum, 2021).

### Normal-weight dyslipidemia

Individuals diagnosed with dyslipidemia are often affected by obesity; however, non-obese individuals with dyslipidemia and metabolic abnormalities constitute another important patient group (Højland Ipsen et al., 2016). The latter condition is also referred to as normal-weight dyslipidemia (NWD) (Højland Ipsen et al., 2016). As feeding a HFD or HSD had no significant effects on lipid metabolism, it is reasonable to assume that they did not induce obesity in Chinchilla rabbits. However, HFSD-fed male Chinchilla rabbits had average weight and BMI but severe dyslipidemia. Thus, they either showed NWD (Højland Ipsen et al., 2016) or metabolically unhealthy normal weight (MUNW) (Badoud et al., 2015). A possible mechanism is that these rabbits have increased levels of HDL. Several studies have also reported that HFSDs induce rabbit NWD. For instance, a HFD (10% corn oil, 8% lard) fed over 1.5 months can induce metabolically obese normal weight (MONW) in male hybrid Flanders rabbits (Alarcon et al., 2018). HFSD (37% sucrose, 10% pork lard) for 6 months (Yin et al., 2002) and HFSD (30% sucrose, 10% fat) for 12 months (Liu et al., 2016) can induce weight loss and dyslipidemia in male New Zealand white rabbits. NWD and/or MUNW affects ∼30% of normal-weight persons globally but its mechanism is still unknown (Klitgaard et al., 2020). Our HFSD-fed Chinchilla rabbits may, therefore, be a suitable animal model for studying this human disease.

### RPD-like lesions and lipid droplets around RPE

The most striking findings in the retina of HFSD-fed Chinchilla rabbits are RPD-like lesions (identified by SD-OCT) and lipid droplets around RPE cells (identified by Toluidine Blue staining and TEM). By using SD-OCT, these lesions were visible in our experiments between the RPE and the ellipsoid zone (photoreceptor segments); they did no display as conventional drusen that have a hyporeflective core and, generally, are located under layers of the RPE. Retinal lesions in our rabbit model appeared − like RPDs − as high-density dome-shaped spots that were hyper-reflective and situated between the ellipsoid layer and RPE (Wightman and Guymer, 2019; Wu et al., 2022). Interestingly, when using TEM, most of these RPD-like lesions were large lipid droplets around RPE cells. Lipid droplets are storage organelles consisting of a hydrophobic core of neutral lipids surrounded by phospholipid monolayer membranes. Although prevalent in adipocytes and liver hepatocytes, they are found in virtually every cell type (Zadoorian et al., 2023). RPE cells produce retinyl ester-containing cytoplasmic storage particles and are referred to as retinosomes (Imanishi et al., 2004; Palczewska et al., 2010); they are structurally identical to lipid droplets (Orban et al., 2011). Retinosome formation depends on lecithin-retinol acyltransferase (LRAT), which is essential for regeneration of the chromophore 11-cis-retinal (Hara et al., 2023; Imanishi et al., 2004). Retinosomes enlarge in Rpe65^−/−^ mice as all-trans-retinyl esters cannot be isomerized, and more retinoids are retained in the retinosomes (Imanishi et al., 2004; Palczewska et al., 2010). RNA sequencing of retinas from our animal model revealed downregulated retinol metabolism genes, including RPE65 and LRAT (Table S1), supporting enlarged lipid droplets that result from dysregulated retinol metabolism of RPE cells. RPE lipid droplets are not only lipid storage organelles but also part of the visual cycle and affect the retinal function. Lipid droplet accumulation can damage the RPE function and is a potential risk factor for AMD (Yako et al., 2022). Clearance of lipid droplets ameliorates the AMD phenotype in mice deficient for APOE (Zhang et al., 2023). Our HFSD-fed Chinchilla rabbits may be a good model for studying the biology of lipid droplets in the retina. Drusen mostly comprise lipids, protein and minerals (Curcio, 2018; Wang et al., 2010), RPDs also have lots of lysolipids (LysoPCs, LysoPEs and LysoPAs) (Anderson et al., 2023). One limitation of our current study is that we did not measure the lipids, proteins and minerals in these RPD-like lesions of the rabbit retina. These RPE lipid droplets are similar to recently described AMD-related subretinal lipid globules (SLG) with SD-OCT (Fragiotta et al., 2023). Both are located between the ellipsoid zone and the RPE−BrM complex, but SLGs are hyporeflective, and RPE lipid droplets in HFSD-fed rabbits are hyper-reflective dome-shaped spots (Fragiotta et al., 2023); the relationship between the two deserves further investigation.

### Retinal degeneration and C3 activation

HFSD-fed rabbits had reduced ERG responses, increased numbers of pyknotic nuclei in the ONL, retinal gliosis and reduced retinal vascular density, all of it features of retinal degeneration. Reduced photoreceptors can cause a reduced ERG response but these enlarged lipid droplets also suggest an impaired visual cycle. Decreased retinal vascular density may be a phenotype of high cholesterol-induced atherosclerosis (Fan et al., 2015). Staining for IBA1 and CD68 suggested that most retinal inflammation pathways are not activated. Indeed, RNA sequencing even suggested suppressed expression of genes involved in the complement cascade (including *Serpinb2*, *Serping1*, *C1s*, *C1r*, *C1qc*, *F2r*, *F3* and *Bdkrb2*) in retinas of HFSD-fed rabbits. We also measured the concentration of complement factor C3 in the eye, as it plays a crucial role in the development of dry AMD (Armento et al., 2021; Demirs et al., 2021). Rabbits on the HFSD diet had decreased levels of C3 in the blood but increased levels in the aqueous humor. Immunostaining of C3 also confirmed the activation of complement cascade proteins in these rabbit retinas. C3 is critical for activating the three complement activation pathways (Sahu and Lambris, 2001) and a therapeutic target for dry AMD (Piri and Kaplan, 2023). Last year, the United States Food and Drug Administration (FDA) approved the first intravitreal injection therapy for geographic atrophy with the C3 inhibitor Pegcetacoplan (Piri and Kaplan, 2023). Systemic C3 levels are associated with weight (Engström et al., 2005). Thus, their reduced blood C3 levels might explain why our HFSD-fed rabbits had average weight. A HFD increased ocular fundus autofluorescence (lipofuscin), suggesting high levels of bis-retinoid in the mouse retina (Kim et al., 2023). Photo-oxidation products of the bis-retinoid N-retinylidene-N-retinylethanolamine (A2E) can activate the complement cascade, including C3 (Kim et al., 2021; Zhou et al., 2006), explaining how HFSD induces increased levels of C3 in rabbit eyes. This seems different to our RNA sequencing data but might only reflect the complicated networks of the complement cascade. Therefore, the relationship between blood and ocular C3 levels might be an interesting topic to follow up in the future.

In conclusion, by using male Chinchilla rabbits as a model system, we made several striking findings. First, HFSD can induce clinical and pathological phenotypes similar to those of dry AMD in humans, including RPD-like lesions, retinal pigment changes and retinal degeneration. Second, we proved that these phenotypes are synergistic effects of feeding a HFSD. Third, HFSD induced severe dyslipidemia but not obesity; thus, this model system is also suitable to study normal-weight dyslipidemia. Fourth, we proved these RPD-like lesions are mainly enlarged lipid droplets around RPE cells; therefore, making this model also suitable for research on lipid droplets in the retina. In the future, adjusting the fat and sucrose contents of the diets, in combination with proteomics and transcriptomics, might clarify the mechanisms of lipid droplet accumulation regarding this rabbit model and help getting a full picture of the relationship between systemic and ocular C3 levels.

## METHODS AND MATERIALS

### Animal housing and husbandry

All procedures used in the animal experiments followed the guidelines of the Association for Research in Vision and Ophthalmology (ARVO) statement for the use of Animals in Ophthalmic and Visual Research, and were approved by the Animal Care Committee of Sichuan University West China Hospital (AUP# 2018008A, Chengdu, China). Male Chinchilla rabbits aged 3-4 months (3.5±0.5 kg) (Dongfang Breeding Co., Pizhou, China) were housed in individual cages and a room with controlled humidity (45±5%) at 22±6°C under 12/12-h light/dark cycle. After acclimation with standard rabbit chow (Keao Xieli Feed CO., LTD., Beijing, China) for 2 weeks, animals were randomly assigned into four groups, comprising 12 animals (i.e. 24 eyes) per group, and fed a regular normal diet (ND, standard rabbit chow) as control, a high-fat diet (HFD) of additional 10% lard and 0.5% cholesterol to standard chow, a high-sucrose diet (HSD) of additional 40% sucrose to standard chow, or a high-fat plus high-sucrose diet (HFSD) of additional 10% lard, 0.5% cholesterol, 35% sucrose to standard chow. All animals were fed *ad libitum* for 6 months.

### Clinical observation and BMI measurements

Animals were observed regularly to check their general condition. The body-growth parameters were recorded from the beginning to the end of the experiment. The body length (distance from the nose to the heel in the lateral decubitus position), height (distance from the acromion in the shoulder to the tip of the paw in the same position), and weight were measured as previously described (Arias-Mutis et al., 2018; Zarzoso et al., 2014). Body mass index (BMI) was calculated as body weight (kg)×[body length (m)×height (m)]^−1^.

### Plasma lipid and lipoprotein analysis

Rabbits were fed the experimental diets over 6 months, during which blood samples were taken after 2, 3, 4 and 6 months. Levels of triglycerides, total cholesterol, low-density lipoprotein (LDL) cholesterol and high-density lipoprotein (HDL) cholesterol were determined using Wako assay kits.

### Fasting glucose test and intravenous glucose tolerance test

Over the 6 months special diet period, blood glucose levels were regularly measured using a glucose meter (Accu-Check Performa, Roche Diagnostics, China), with samples taken after overnight fasting at the end of month two, three, four and six. Fasting glucose test and intravenous glucose tolerance test (IVGTT) were performed using the previously described method (Zhang et al., 2001). In brief, after overnight fasting, all rabbits were intravenously injected with glucose solution (0.6 g/kg of body weight) through the marginal ear vein, and blood samples were taken via the auricular artery at 0, 30, 60, 120 and 180 min later. The plasma glucose area under the curve (AUC) was calculated using the mean glucose level and the time using GraphPad Prism 8. AUC is an index of whole glucose excursion after glucose loading.

### Measuring blood and ocular levels of complement factor C3

Blood samples were collected from the marginal ear vein of rabbits, and the aqueous humor (∼150 µl) was collected using a 30-gauge disposable needle and insulin syringe at the temporal limbal region (Wang et al., 2021). The plasma and humor samples were placed on ice during the procedure and stored at −80°C until analysis. Samples were analyzed by the Luminex 200 Analyzer (xMAP^®^ Technology, Luminex Co., Austin, Texas, USA). Levels of C3 in the plasma and the aqueous humor were determined using ELISA kits (E08668Rb, CUSABIO®, Wuhan, China) following the manufacturer's protocol. In brief, 100 µl diluted samples in the dilution buffer in the ELISA kit were incubated within the well for 2 h at 37°C, the secondary antibodies for 2 h at RT, and streptavidin phycoerythrin reagent for 30 min at RT. Samples were read on the Luminex 200 system. Tests were run in duplicate for each sample. The concentration was determined by comparing it with a standard curve.

### Ocular multimodal imaging and ERG recording

Multimodal ocular imaging was performed every month during the experimental period. All eyes underwent pupillary mydriasis induced with 0.5% tropicamide and 0.5% phenylephrine hydrochloride (Mydrin-P, Santen Pharmaceutical, Osaka, Japan). Before imaging, animals were anesthetized with intravenous injection of 2.5% soluble sodium pentobarbital. Color fundus photographs (CFP) were taken by a TRC-50DX retinal camera (Topcon, Japan). Multicolor (MC) fundus photography, fundus fluorescein angiography (FFA) and spectral domain-optical coherence tomography (SD-OCT) images were taken by using the SPECTRALIS HRA+OCT diagnostic imaging platform (Heidelberg Engineering, Heidelberg, Germany). MC photographs had three wavelengths (518 nm, 486 nm, and 815 nm) to generate three retinal images. The field of view was 30×30 degrees and the resolution 768×768 pixels. The images were magnified using the built-in software to evaluate the retinal lesion following the previously described method (Graham et al., 2018; Wang et al., 2021) FFA photographs were taken after 10% sodium fluorescein was injected into the marginal ear vein at a dose of 0.05 ml/kg and a rate of 1 ml/s. Both early-phase (30 s) and late-phase (5-6 min) FFA images were recorded. The built-in software of SD-OCT scans with a 30°×30° region centered on the optic nerve head and consisting of 49 B-scans, each with 1024 A-scans and 25 frames averaged. OCT was applied to evaluate RPD-like lesions (Badal et al., 2018; Klein et al., 1991).

At the end of the study, ERGs were recorded as previously described (Wang et al., 2021). In brief, rabbits were anesthetized with intravenous injection of pentobarbital (25 mg/kg). After anesthesia and pupil dilation, the animals had bipolar electrodes placed on the cornea of both eyes and the reference and ground electrodes inserted into ears (model E5; Grass Technologies, West Warwick, RI). The Full-field ERG was recorded using a RETI-PORT 21 Compact (Roland Consult, Brandenburg an der Havel, Germany) following the International Society for Clinical Electrophysiology of Vision (ISCEV) guidelines (Robson et al., 2022). Scotopic ERGs were recorded in the dark after 30 min of dark adaptation, photopic ERGs were recorded after 10 min of light adaptation. The same single full-field flashes and filtering were used for scotopic and photopic ERG. Full-field white light flashes were applied for the 30 Hz flicker ERG recording. Amplitudes and peak latencies were analyzed following the established method (Wang et al., 2021).

### Histopathology, immunohistochemistry, TUNEL staining and whole-mount staining

For necropsy, rabbits were euthanized with intravascular injection of pentobarbital sodium, the eyeballs from each group were collected for histopathology and immunohistochemistry examination. After rinsing in isotonic sodium chloride solution, a 5-mm diameter window near the limbus wall of globes was opened to permit the fixative into the eyeball. The eye was then placed in a modified fixative containing 2% glutaraldehyde and 2.5% formaldehyde in 0.1 M cacodylate buffer at 4°C. After 24–48 h fixation, the anterior segment was removed, and the posterior cup was cut into half eyecups. For hematoxylin and eosin (H&E) staining, half-eyecups were embedded in paraffin, sectioned (3 μm in thickness), and stained with H&E for histopathological examination.

For the immunohistochemistry study, half-eyecups were frozen and processed into sections (12 μm). Sections were first incubated in blocking solution (5% normal donkey serum, 0.1% Triton X-100 in 1× PBS) for 1 h, followed by incubation with primary antibodies against complement C3 (Abcam, Ab90814, mouse monoclonal, 1:50 dilution), CD68 (Abcam, Ab201440, Mouse monoclonal, 1:100 dilution), Allograft inflammatory factor 1 (IBA1, also known as AIF1; FUJUFILM Wako, 019-1974, rabbit polyclonal, 1:500 dilution) and glial fibrillary acidic protein (GFAP; Abcam, Ab7260, rabbit polyclonal, 1:1000 dilution) at 4°C overnight. Subsequently, slides were incubated with corresponding secondary antibodies conjugated to Alexa-Fluor-488 or Alexa-Fluor-568 (Invitrogen, USA) for 1 h at RT in the dark. Finally, slides were counterstained with 4'6-diamidino-2- phenylindole (DAPI; Sigma Aldrich Corp.) and mounted with Mowiol mounting medium. The negative control was performed by replacing primary antibodies with PBS.

To detect apoptotic cells within rabbit retinas, we performed TUNEL (Terminal dUTP nick end-labeling) staining (ApopTag^®^ Peroxidase in Situ Apoptosis Detection Kit, Cat. No S7100, Millipore, Temecula, USA) according to the manufacturer's protocol. Briefly, paraffin retinal sections were deparaffinized and immersed in 1xPBS for 15 min. After hydration, these sections were permeabilized with proteinase K for 5 min and then incubated with 3% hydrogen peroxide (H_2_O_2_) to inactivate endogenous peroxidases. After that, the TdT enzyme and TdT labeling reaction mix was added, and incubated for 60 min at room temperature. Then, the reaction was stopped by adding the stop buffer at 37°C for 10 min. The sections were incubated with a streptavidin-HRP conjugate for 30 min at room temperature to determine the biotinylated DNA. Subsequently, they were washed with 1XPBS, diaminobenzidine (DAB) substrate was added, and the reaction was stopped. The slides were then counterstained with hematoxylin, dehydrated, cover-slipped, and observed under a light microscope.

For whole-mount staining, retinas from half-eyecups were isolated. They were incubated with biotin-conjugated *Bandeiraea simplicifolia* isolectin B_4_ to stain vascular endothelial cells (IB4 1:200, Sigma, L2140) for 1 day at 4°C, then with Streptavidin Alexa Fluor 568 for 2 h at 4°C. After brief washes with PBS, radial cuts were made to divide the retina into two quadrants to flatten the retina, and the tissue was mounted with Mowiol. For vascular blood vessel analysis, representative images were analyzed using AngioTool software (https://ccrod.cancer.gov/confluence/display/ROB2/Home) to assess the vessel covered area (%), average vessel length, and mean E lacunarity of the vascular plexus. In brief, measured were three images per eye and seven half-eyecups from the same group [320×320 μm field of view (FOV) at ×200 magnification].

### Transmission electron microscopy (TEM) and Toluidine Blue staining

From each group, eyeballs from different animals were collected for TEM examination and Toluidine Blue staining. Eyes were prefixed with 3% glutaraldehyde injected intravitreally through the pars plana, followed by immersive fixation in 2% glutaraldehyde in 0.1 M phosphate buffer at pH 7.4 and 4°C for a minimum of 12 h. Then, they were incubated with 3% EDTA solution for 20 min. A piece of eye-cup tissue (∼2×2 mm) was cut from the peri-optic disc region guided by multimodal images to include an area with the highest density of drusen-like lesions. The tissues were post-fixed in 1% osmium tetroxide in 0.1 M cacodylate buffer for 2 h at 4°C. Tissues were dehydrated using a series of acetone and then embedded with epoxy resin (Epon 812). Semi-thin sections (1.0 µm) were stained with Toluidine Blue. The ultra-thin sections (50 nm) cut with an ultra-microtome (EM UC7, Leica, Germany) were treated with 2% uranyl acetate in water and lead citrate for contrast and were examined by JEM-1400PLUS transmission electron microscopy (TEM; JEOL Ltd., Tokyo) with an image magnification between 4800× and 5800×.

### RNA-sequencing

Total RNAs were extracted from dissected rabbit retinas using TRIzol (Thermo Fisher) and treated with RNAse-free DNAse I (New England Biolabs) to remove genomic DNA. The yield of total RNA was assessed using NanoDrop Microvolume Spectrophotometers (NanoDrop Technologies). The cDNA libraries were prepared using an Illumina TruSeq RNA sample preparation kit, and the quality was assessed using an Agilent 2100 Bioanalyzer (Agilent Technologies). For sequencing, the cDNA libraries were loaded on an Illumina HiSeq 2500 at Biomaker (Beijing, China). The raw sequence reads in FASTQ format were processed and analyzed as previously reported (Gregorieff et al., 2015; Liu et al., 2014). Briefly, sequencing quality was first assessed using FastQC, and poor-quality 5′-end of reads were trimmed using a Perl script and then mapped onto the rabbit genome using TopHat2, allowing for up to two mismatches as default settings. Reads mapped onto multiple genomic locations were discarded, and a custom R script was used to calculate fragments per kilobase million (FPKM) of each gene and obtain the expression profile of each sample. The expression-fold change of each protein-coding gene of retinas obtained from a specific diet group compared with retinas obtained from the control group was calculated using the following formula: Fold-change=(FPKM^diet^+1)÷(FPKM^control^ +1). Genes for expression-fold changes of >1.54 or <0.65 were selected as differentially expressed genes (DEGs). Function enrichment (KEGG and Gene Ontology) of DEGs was performed using Enrichr (https://maayanlab.cloud/Enrichr/) (Kuleshov et al., 2016); pathways with a *P*<0.05 were chosen to report.

### Sample size, image and statistical analyses

We chose the sample size based on power analysis and common sense in rabbit/diet experiments (Arias-Mutis et al., 2017; Festing, 2018; Liu et al., 2016). Sample sizes (*n*) regarding numbers of rabbits and diet experiments were calculated using the equation *n*=[2 (Z_α_+Z_1–β_)^2^]/SES^2^ (Bagiella and Chang, 2019; Festing, 2018; Kadam and Bhalerao, 2010), with Z being the constant (set by convention) according to power of the study, and with α (type I error) = 0.05; β (type II error) =10–20% and a two-sided effect. The standardized effect size (SES; also known as Cohen's *d*) indicates the magnitude of the studied effect, i.e. the magnitude of the difference between the means of two groups in units of standard deviations (s.d.), and is equal to the effect size (ES) divided by the pooled s.d. (Festing, 2018). For laboratory animal experiments, it has been suggested that an SES of 1.1, 1.5 or 2.0 SDs represent small, moderate or significant treatment responses, respectively (Festing, 2018). Therefore, long-term diet treatment may have mild (HFD or HSD) to substantial (HFSD) effects on rabbits; according to the formula mentioned above, between four and seven eyes are required to detect its impact with a 80% power, or between six and ten eyes are needed to detect its effects with a 90% power, in our experiments. Since rabbits can die during these experiments, we used 12 rabbits per diet group at the beginning of the experiment.

Cells positively stained with antibodies against C3, CD68 or IBA1 and with Toluidine Blue were observed by using the Zeiss Axio Imager Z2 microscope and Nikon A1RMP confocal microscope. Positively stained cells (C3^+^ in Fig. 5D, IBA1^+^and CD68^+^ in Fig. 7D) were counted manually with Image J 1.50b and a cell counter plugin (https://imagej.nih.gov/ij/). The minimal number of immunostaining images for each group was 28 (two images per section, two sections per retina, seven half-retinas). All images for cell counting were captured under a fluorescence microscope using a 20×objective lens.

Number, diameter and area of RDP-like lesions were counted, and measured by using Image J 1.50b and a cell counter, and diameter/area measurement plugin. The minimal number of H&E images for each group was 28 (two images per section, two sections per retina, seven half-retinas) when counting the number of lesions, the minimal number of RPD-like lesions for size and area measurements was 50 per group.

Quantitative variables of this study are presented as mean±standard deviation (Mean±s.d.). Statistical analysis was performed using the GraphPad Prism (GraphPad Prism Software, Inc., San Diego, CA, USA). Results were analyzed by one-way analysis of variance (ANOVA) followed by Bonferroni correction for multiple comparisons and unpaired Student's *t*-test for comparisons between two groups. *P*<0.05 was considered statistically significant.

## Supplementary Material

10.1242/dmm.052015_sup1 Supplementary information

Table S1. List of DEG levels in rabbits fed a HFD, HSD or HFSD versus ND-fed rabbits.
