# Supplementary material for: A high-fat plus high-sucrose diet induces age-related macular degeneration in an experimental rabbit model
Source: Dis Model Mech. 2024 Nov 27;17(11):dmm052015. doi: 10.1242/dmm.052015 (PMC11625886; doi:10.1242/dmm.052015)
Supplement: Supplementary information [file dmm-17-052015-s1.pdf]

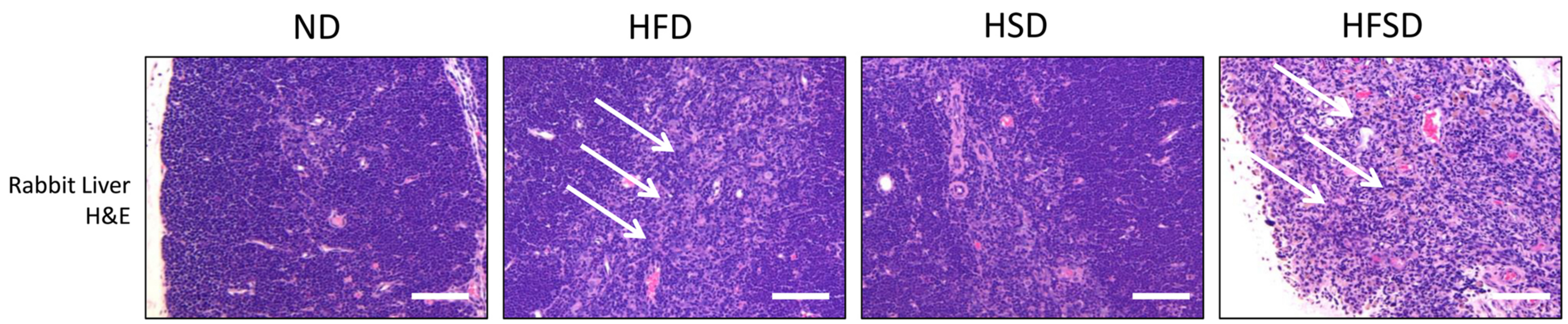

Fig. S1. H&E staining images of livers of male Chinchilla rabbits after feeding with indicated diets for six months. Liver fibrosis is indicated by white arrows. ND, normal diet; HFD, high-fat diet; HSD, high-sucrose diet; HFSD, high-fat and high-sucrose diet. Scale bar is 200 µm.

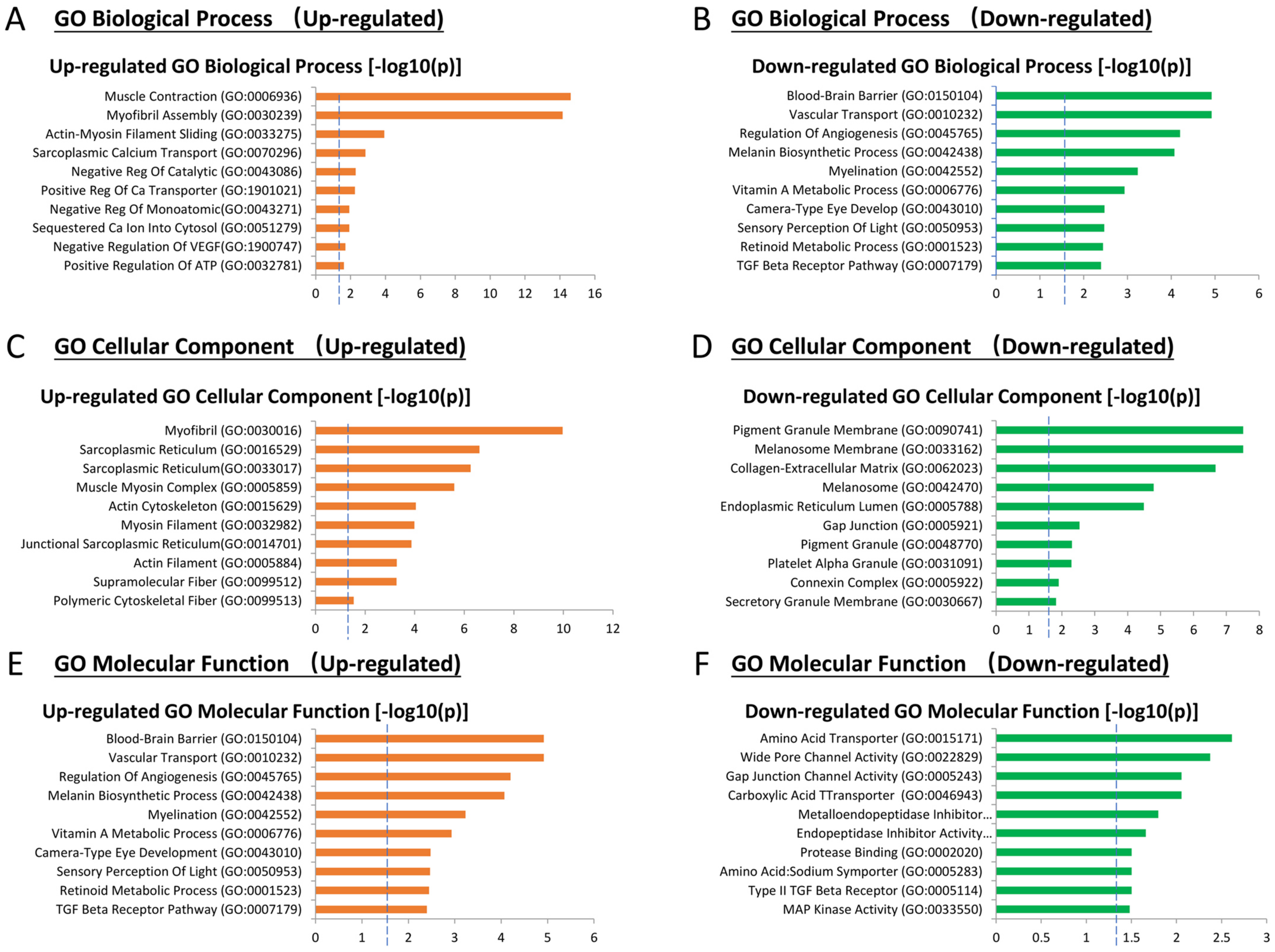

Fig. S2. Gene Ontology (GO) analyses of DEGs of rabbit retinas after feeding with HFD, HSD and HFSD for six months. Analysed were up-regulated (A) and down-regulated (B) GO Biological Processes, up-regulated (C) and down-regulated (D) GO Cellular Components, and up-regulated (E) and down-regulated (F) GO Molecular Functions. Dashed lines indicate P<0.05.

Disease Models & Mechanisms • Supplementary information

Table S1. List of DEG levels in rabbits fed a HFD, HSD or HFSD versus ND-fed rabbits.

Available for download at
https://journals.biologists.com/dmm/article-lookup/doi/10.1242/dmm.052015#supplementary-data
